# Enlargements of the Spleen—I

**Published:** 1908-07-25

**Authors:** 


### Medicine.

### ENLARGEMENTS OF THE SPLEEN—I.

Enlargements of the spleen and their differential diagnosis form a very important chapter in practical medicine, and the following is a list of conditions in which the spleen may be of unusual size:—

A. *Very Great Enlargement.*—Spleno-medullary leuchæmia; some cases of chronic malaria; kala-azar; some cases of cirrhosis of the liver, Hodgkin's disease, splenic anæmia, infarction in infective endocarditis, and pseudo-leuchæmia infantum.

B. *Considerable Enlargement.*—Malaria; cirrhosis of the liver; lymphatic leuchæmia; Hodgkin's disease; splenic anæmia; infective endocarditis, infarction from causes other than infective endocarditis. Some cases of pernicious anæmia, of lardaceous disease, of congenital syphilis, and of rickets.

C. *Slight Enlargement.*—Typhoid fever; less commonly the other specific fevers; some cases of malaria, cirrhosis of the liver, some cases of Hodgkin's disease, of pernicious anæmia, of lardaceous disease, of infective endocarditis, and of rickets; thrombosis of the portal vein; pressure upon portal vein by adjacent tumours, *e.g.* of the liver, gall-bladder, pancreas, stomach; obstruction to the inferior vena-cava above the hepatic veins by thrombosis or from filtration by a mediastinal tumour; chronic lung disease, such as fibrosis; a few cases of chronic heart disease. The above long list takes no account of pathological rarities such as primary or secondary growths in the spleen, gumma, hydatid, abscess, or caseous tubercle of the spleen, or traumatism with rupture beneath the capsule.

### Physical Signs of Enlarged Spleen.

The first step in the examination of an abdominal lump is to determine whether or not it conforms with the physical signs of enlargement of the spleen, which are as follows:—

I. *Inspection.*—(*a*) If the organ is only slightly or moderately enlarged there is no alteration in the size or shape of the abdomen.

(*b*) If it is considerably or enormously enlarged the abdomen may be much distended, and at a first glance the distension may appear to be uniform, as in ascites. A closer inspection, however, may show that it is by no means uniform, there being a distinct bulging on the left side especially in the left hypochondriac and left half of the umbilical regions.

(*c*) In some cases the inner border is tilted forwards and a distinct ridge or edge can be seen, pushing forwards the abdominal wall and running downwards and inwards from the left costal margin about the anterior axillary line towards the umbilicus. This is particularly the case when the liver is also much enlarged, so as to tilt the spleen.

(*d*) In a few cases a distinct notch can be seen in this edge or ridge, but the abdominal parietes need to be thin for this to be possible.

(*e*) When the patient takes a deep breath a distinct downward movement of the prominence which causes the bulging of the abdominal wall may be detected.

(*f*) The spleen may be so enormously enlarged that its lower end is jammed into the pelvis, so that when a deep breath is taken no downward movement is possible.

II. *Palpation.*—(*a*) If the enlargement is slight the spleen may not be felt on palpating the abdomen unless with the flat hand against the abdominal wall the tips of the fingers are placed close underneath the left costal margin behind the left nipple line, and the patient is directed to take a deep breath. The observer's hand should be kept almost still, all the movement being performed by the patient; it is of great assistance to use the other hand as a firm support to the lower left ribs posteriorly, in order to keep the spleen as far forward as possible. Either a firm mass with a distinct edge, or else an indefinite sense of increased resistance, may be felt descending against the fingers as the patient's diaphragm pushes it down. Occasionally the organ is so soft that it cannot be felt, though a post-mortem examination may prove it to have been enlarged. This is sometimes the case in typhoid fever, and often in septic states.

(*b*) If the enlargement is moderate a tumour may be felt on the left side of the abdomen, coming down from the thorax to the abdominal parietes.

(*c*) If the enlargement is enormous, a large mass which is usually smooth and firm may be felt extending from the left costal margin to the umbilicus, crossing the middle line at this level and sometimes reaching as far over as the right iliac fossa and as low down as the left Poupart's ligament.

(*d*) If there is some other abdominal tumour in addition to the spleen—a large liver, for example—the spleen may be prevented from crossing the middle line just below the umbilicus as it naturally tends to do, and it may be confined to the left half of the abdomen.

(*e*) The tumour will be found to be closely approximated to the abdominal wall.

(*f*) It will be felt to come down so close under the costal margin that it will be impossible to place the hand between it and the costal margin, and to define its upper limit by palpation. There are rare exceptions to this rule, notably in cases of dislocated or wandering spleen; the latter has been displaced even as low as the pelvis, and naturally the diagnosis is then extremely difficult.

(*g*) A well-defined, hard, slightly-rounded, or fairly sharp edge may be felt beginning between the ensiform cartilage and the left nipple line and passing downwards and to the right towards the umbilicus.

(*h*) A well-defined notch, or in some cases two or three, may be felt if the inner border is carefully felt.

(*i*) If the hand is placed over the lower pole of the tumour and the patient then takes a deep breath, the mass may be felt to move downwards.

(*j*) If the tumour is palpated bimanually it will be appreciated that posteriorly the loin is flaccid and not filled out, and that the mass is lying close under the abdominal wall in front.

(*To be continued.*)

